# Domestic Summary

**Published:** 1839-02-16

**Authors:** 


### DOMESTIC SUMMARY.

*American Phrenological Journal.*—We continue to receive this journal, published monthly by A. Waldie. It has our good wishes for its success.

---

The American Philosophical Society has declined acceding to the proposal which emanated from Boston, (noticed in No. 2,) relative to an *American Institution for the Cultivation of Science.*

---

*Lithotomy in the South.*—Dr. Antony, one of the ablest and most experienced Southern surgeons, states in a late number of the Southern Medical and Surgical Journal, that he "knows of no cases of distress from urinary calculi having occurred in his section of country, so serious as to call for surgical aid, at least for extraction from the bladder—and of but one instance in the whole population of Augusta, (Georgia,)

and the surrounding country, of a person for whom that operation has been demanded; and that case occurred many years ago." We noticed, in a recent number, the *hundred and fifty-seventh* operation for lithotomy, of Dr. Dudley, of Kentucky.

---

*Foreign body in the Esophagus six weeks—its extraction.*—A boy of about five years of age, the son of Mr. J. L., of Augusta, was playing with a large ivory button in his mouth, when he accidentally swallowed it. Presuming it had passed into his stomach, no notice was taken of the event until meal-time, when the boy complained that he could swallow nothing but liquids, and that even these occasioned pain in the esophagus, opposite the upper extremity of the sternum. It was now presumed that the button had lodged in this part of the passage, and a physician was called, who introduced without difficulty an elastic tube into the stomach, without detecting any thing indicative of the presence of the foreign body. The presumption was, that the painful deglutition was to be attributed to abrasion of the mucous surface, and the boy was ordered to use liquid and unirritating food. This state of things continued for several weeks, during which time the tube was again passed without obstacle into the stomach—emetics, I believe, were administered, &c.

At the end of six weeks, Dr. Dugas was called, and, on examination, detected the presence of the button at the seat of pain, and withdrew it with a common probang. It was of ordinary thickness, and measured one inch in diameter. No unpleasant effect followed its removal, and the soreness soon subsided.

*Remarks.*—This case presents the remarkable fact, of the presence, during six weeks, of a foreign body in so delicate a texture as that of the esophagus, without occasioning any serious inflammation, and without disturbing the general health. It was so low down as not to be felt by external pressure, and the button having no *eye*, permitted the free passage of the small tube.—*Professor Dugas' Surgical Cases, Southern Med. and Sur. Jour.*

---

*Observations on the Carbonate and Protomuriate of Iron.* By Wiliam Procter, Jr.—The particular object of this notice is to call the attention of pharmaceutists to the fact, that the carbonate of iron, obtained by M. Vallet's method, can be applied in the process for making several ferruginous preparations, with a certainty and accuracy that will go far to recommend its use.

*Tincture of Muriate of Iron.*—The formula of the United States Pharmacopœia consists in acting on the precipitated carbonate of iron with muriatic acid, and afterwards adding a given quantity of alcohol. Owing to the variable nature of the precipitated carbonate, as respects the proportion of protoxide of iron which it contains, we have a preparation varying in strength; besides, a portion of uncombined acid remains, which reacts on the alcohol, as is evident from its etherial odour.

The process now offered is to take any quantity of officinal muriatic acid, and saturate it with Vallet's carbonate of the protoxide of iron; by these means, a solution of the protomuriate of iron is obtained, which contains a quantity of the ferruginous salt in proportion to the strength of the acid employed. To this solution as much alcohol is to be added as will reduce the preparation to the proportion of thirty-two grains of the protoxide, or about sixty-four grains of the protomuriate, to the ounce of tincture.

In this state, however, there is a continual liability to the absorption of oxygen, by which a portion of the proto- is converted into permuriate, and to the separation of peroxide of iron. To remedy this inconvenience, a portion of honey is to be added to the muriatic solution at the time of its mixture with the alcohol. This saccharine substance protects the protosalt, at the same time that it does not interfere with the chemical or therapeutic properties of the medicine.

In making the muriatic solution, take 480 grains of muriatic acid, of sp. grav. 1.16, which contains 32.32 per cent. of acid, and saturate it with protocarbonate of iron: consequently, for every thirty-seven grains of muriatic acid, thirty-six grains of protoxide of iron are required: so that $32.32 \times 4.80 = 154.836$ of acid, then as 37 : 36 :: 154.836 ·· 150.651, the quantity of protoxide of iron required to the ounce of muriatic acid. Owing to the moist state of the carbonate, when used, the amount of liquid, after saturation, is just double. Whatever may be its bulk, however, it contains about 150 grains of protoxide of iron, so that the other fluid must be added till the solution contains thirty-two grains of the protoxide to the ounce.

The principal advantage of this process is, that a given amount of acid requires a much larger portion of iron for its saturation than when the peroxide is used, because the permuriate is a sesquimuriate. Thus, $(3\ Fe.O) + (3\ Cl.H) - (Fe^2O^3) + (3Cl.H) = Fe.$ 1 equivalent; consequently, in saturating three equivalents of muriatic acid with the protoxide and peroxide respectively; there is one whole atom, or fifty per cent. of iron, more in the former than in the latter; hence the muriate of the protoxide is decidedly to be preferred.

The following is a formula for tincture of muriate of iron:

| | |
|---|---|
| ℞.—Acid. Hydrochlor. | ℥ij. (troy.) |
| Ferri Protocarb. | q. s. ad saturand. |
| Mellis, | ℥ijss. |
| Alcoholis, | q. s. |

Saturate the acid with the carbonate, then add the honey, and finally, sufficient alcohol to make nineteen fluid ounces of tincture. After standing six hours, filter for use.

Carbonate of iron can be advantageously employed in making the tartrate and acetate of iron, and the tartrate of iron and potassa.—*Am. Journal of Pharmacy, January*, 1839.

